# High-Resolution *In Vivo* Imaging of Regimes of Laser Damage to the Primate Retina

**DOI:** 10.1155/2014/516854

**Published:** 2014-05-07

**Authors:** Ginger M. Pocock, Jeffrey W. Oliver, Charles S. Specht, J. Scot Estep, Gary D. Noojin, Kurt Schuster, Benjamin A. Rockwell

**Affiliations:** ^1^The U.S. Air Force Research Laboratory, 711 HPW**/**RHDO, 4141 Petroleum Road, JBSA Fort Sam Houston, TX 78234, USA; ^2^Department of Biomedical Engineering, The University of Texas at Austin, University Station No. C0800, Austin, TX 78712, USA; ^3^Penn State Hershey Anatomic Pathology, 500 University Drive, Hershey, PA 17033, USA; ^4^U.S. Army Veterinary Corps at the Tri-Services Research Laboratory, 4141 Petroleum Road, JBSA Fort Sam Houston, TX 78234, USA; ^5^TASC Inc., Biomedical Sciences and Technologies Department, JBSA Fort Sam Houston, TX 78234, USA

## Abstract

*Purpose*. To investigate fundamental mechanisms of regimes of laser induced damage to the retina and the morphological changes associated with the damage response. *Methods*. Varying grades of photothermal, photochemical, and photomechanical retinal laser damage were produced in eyes of eight cynomolgus monkeys. An adaptive optics confocal scanning laser ophthalmoscope and spectral domain optical coherence tomographer were combined to simultaneously collect complementary *in vivo* images of retinal laser damage during and following exposure. Baseline color fundus photography was performed to complement high-resolution imaging. Monkeys were perfused with 10% buffered formalin and eyes were enucleated for histological analysis. *Results*. Laser energies for visible retinal damage in this study were consistent with previously reported damage thresholds. Lesions were identified in OCT images that were not visible in direct ophthalmoscopic examination or fundus photos. Unique diagnostic characteristics, specific to each damage regime, were identified and associated with shape and localization of lesions to specific retinal layers. Previously undocumented retinal healing response to blue continuous wave laser exposure was recorded through a novel experimental methodology. *Conclusion*. This study revealed increased sensitivity of lesion detection and improved specificity to the laser of origin utilizing high-resolution imaging when compared to traditional ophthalmic imaging techniques in the retina.

## 1. Introduction

The expansive incorporation of laser technology into an array of medical, commercial, and military applications has introduced a multitude of laser based technology developments [1]. The medical laser industry is projected to surge over the next decade [2] and promises to deliver an array of commercial and clinical devices for the diagnostic evaluation and treatment of disease. The continually expanding use of laser technology requires careful monitoring of the laser safety standards with a full understanding of the mechanisms of damage and adequacy of the data supporting the standard. This is especially highlighted in the ophthalmic community with regard to a study by Morgan et al. [3]. Using an adaptive optics (AO) confocal scanning laser ophthalmoscope (AO cSLO), they observed permanent change to the retinal pigment epithelium (RPE) from laser exposure an order of magnitude lower than previous experimental data which served as a base for current laser safety standards [4]. Their results underscore the need for the use of high-resolution imaging as an additional endpoint for evaluating retinal laser exposures for the establishment of laser safety standards. The ANSI standard [5] sets the maximum permissible exposure (MPE) as the level of laser radiation to which an unprotected person may be exposed without adverse biological changes in the eye or skin. The standard is based upon* in vitro* [6] and* in vivo* [7–11] laser bioeffects experiments designed to determine the limits of tissue to laser damage as well as to describe the photobiological processes involved [12]. Experiment exposure parameters are based on the mechanisms governing light induced tissue damage: power density, total exposure duration, and wavelength of irradiating energy. With respect to the eye, the wavelength dependent optical properties of the anterior segment and vitreous also play an important role. Retinal damage from laser or noncoherent light sources can occur via three principal light damage mechanisms: photothermal, photochemical, and photomechanical damage [13].

Retinal laser damage studies have traditionally used a direct-view funduscope (ophthalmoscope) to observe changes to retinal tissue following laser exposure. These studies have been limited by the resolution limits of the funduscope to resolve lesion formation in addition to lack of cross-sectional views necessitating histology and subsequent euthanasia of the subject. More recently, commercially available high-resolution retinal devices such as spectral domain optical coherence tomography (SD-OCT) have been incorporated into studies of laser induced damage to the retina for laser safety recommendations [14, 15] and to monitor the healing response of retinal laser treatments for clinical applications [16–18].

Since the transition of adaptive optics (AO) technology from astronomy to ophthalmology almost 17 years ago, AO has made significant progress in the performance of ophthalmic instruments. To date, there have been no studies utilizing AO SD-OCT to evaluate laser injury to the retina nor the healing response. The work presented here is intended to facilitate laser lesion detection and identification. In this pilot study, the progression of photomechanical and photothermal/photochemical laser exposure to the retina of the nonhuman primate (NHP) was documented using a combined AO confocal scanning laser ophthalmoscope (AO cSLO) and an AO SD-OCT. Real-time imaging of lesion development and characterization over time will enable better understanding of regimes (thermal, photochemical, and photodisruptive) of laser damage in the primate retina. The AO SD-OCT and AO cSLO provide researchers with high-resolution images of retinal layers to investigate laser damage* in vivo* and an opportunity to observe laser damage over multiple time points without the need for histopathology as a single end point for observation.

## 2. Methods

### 2.1. Primate Preparation

Sixteen eyes of eight male cynomolgus (*Macaca fascicularis*) monkeys between the ages of 6 and 12 years (4.4–7.0 kg) were used for this experiment only. All subjects were used for* in vivo* imaging and histological study of laser exposure to the retina. Prior to retinal imaging, subjects were restrained utilizing 2–6 mg/kg intramuscular (IM) injection of Telazol. Once restrained, two drops each of proparacaine HCl 0.5%, phenylephrine HCl 2.5%, and tropicamide 1% were administered to both eyes. Additional drops were administered throughout the procedure as needed to maintain a dilated pupil. Once initial sedation was achieved with Telazol, lactated Ringer's solution (10 mL/kg per hour flow rate) and propofol were introduced with placement of intravenous (IV) catheters in the saphenous veins of the lower limbs. An initial dose of propofol (5–12 mg/kg) was followed by a continuous maintenance dose of 0.2–1.0 mg/kg/min as required to maintain the desired plane of anesthesia. Prior to prone placement of the subject on a 5-axis goniometric translation stage, the subject was intubated, and a peribulbar injection of 4% lidocaine was administered in the first eye to be imaged to reduce extraocular muscular movement. Additional peribulbar injections were administered as required reducing extraocular movement prior to imaging. The subject's temperature, oxygen saturation, and pulse were continuously monitored throughout the experimental protocol. Body temperature was maintained by the use of a temperature management unit (Bair Hugger, Arizant Healthcare Inc., Eden Prairie, MN) in conjunction with blankets. Prior to each imaging session, axial length, used to scale images, was measured (PalmScan AP200, Mico Medical Devices). Primate's eyelids were held open with a wire lid speculum for each imaging session and a hard permeable contact lens was used to prevent corneal drying. All experimental procedures were approved by the Brooks City Base Institutional Animal Care and Use Committee and conformed to all United States Department of Agriculture (USDA), National Institutes of Health (NIH) guidelines, and the ARVO Statement for the Use of Animals in Ophthalmic and Vision Research.

### 2.2. Histological Preparation and Light Microscopy

Monkeys were euthanized under deep anesthesia and transcardially perfused with a fixative solution (pH 7.4) consisting of 10% formalin and 2% paraformaldehyde in 0.1 M sodium phosphate buffer. Each animal was sedated with a dose of Telazol (2–6 mg/kg) and then brought to a surgical plane of anesthesia with the use of propofol. Once this plane was achieved, euthanasia was accomplished with an overdose of pentobarbital sodium (10 mg/kg). After each perfusion was complete, eyes were enucleated and immediately were fixed in 10% neutral buffered formalin. A 360° incision was made at the ora serrata and the anterior eye structures were removed from the posterior eye cup. A notch suture was placed in the nasal sclera before enucleation to maintain proper orientation. After fixation, the caudal retina including the optic nerve, fovea, and injury sites was prepared as a flat-mount in paraffin blocks. Five to seven *μ*m serial sections of the retina were placed on positively charged glass slides and stained with hematoxylin and eosin stain on an automated stainer. Sections were evaluated by a board certified veterinary pathologist. Images were taken at 400X with an Olympus DP72 camera (Center Valley, PA) attached to an Olympus BX51 microscope.

### 2.3. Laser Exposure

Three types of laser exposure parameters were delivered to the retina to examine photomechanical and photothermal/photochemical light interactions. Up to 25 lesions were administered to each retina. All laser exposure energies were measured at the cornea. A beam splitter was used to reflect part of the beam to a reference energy meter to determine the ratio of the output beam energy to the energy delivered to the subject. The laser exposure parameters and subjects used for each experiment are summarized in Tables 1 and 2, respectively. Retinal exposure spot sizes were approximated using an ABCD propagation method [19] under the assumption of a collimated 3 mm (1/*e* ^2^) diameter at the corneal plane. Retinal irradiance was calculated based on the assumption of 100% ocular transmission at all exposure wavelengths.

**Experiment 1 (Pulsed Photothermal Regime: Microsecond Pulsed Exposure)**

Five eyes from three primates were exposed to single and multiple pulse 555 nm (Candela LFDL-8, Candela Laser Corporation, Wayland, MA) 2 *μ*s exposures to the macular region with an estimated retinal spot size of diameter of either 120 *μ*m (*n* = 3) or 40 *μ*m (*n* = 2). Retinal exposure energies ranged from 72 to 4,536 mJ/cm^2^.

**Experiment 2 (Photomechanical Regime: Picosecond Pulsed Exposure)**

A Nd:YAG regenerative amplifier (Spectra Physics GCR-3RA, Newport Corp., Irvine, CA) was used to produce 532 nm, 40 ps exposures. The beam was directed through a half-wave plate and polarizing beam splitter cube combination to control the energy delivered to the subject. Retina test sites received laser exposures for energies ranging from 6.2 to 1,843 mJ/cm^2^ (24 *μ*m retinal spot size).

**Experiment 3 (Photochemical/Photothermal Regime)**

Seven eyes from four primates were exposed to collimated 413 nm (105 *μ*m retinal spot size) Krypton ion laser (Coherent Sabre, Coherent Inc., Santa Clara, CA) for 20 seconds in the macular region to investigate photochemical/photothermal interactions. Retinal irradiance exposures ranged from 40.4 to 7,000 J/cm^2^.

### 2.4. AO SD-OCT/cSLO

**Figure 1:**
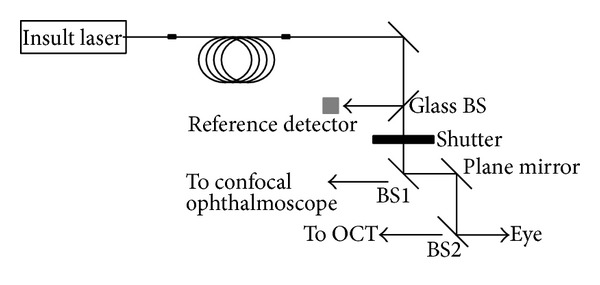
Exposure laser setup and beam introduction into imaging system.

**Figure 2:**
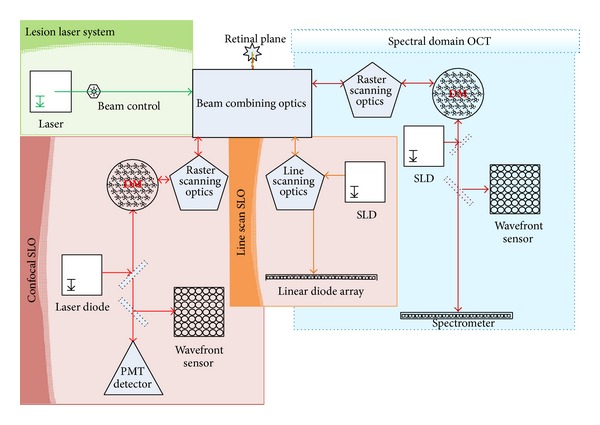
Combined AO cSLO and AO SD-OCT imaging system with wide field line scanning laser ophthalmoscope for collecting complimentary high-resolution images of retinal cross-sections and* en face* field of view.

For most of the experiments, the insult beam was passed through a safety shutter prior to coalignment with the combined AO retinal imager to enable real-time viewing of laser exposure (Figure 1). An AO SD-OCT (Physical Sciences Inc., Andover, MA) with a wide field line scanning laser ophthalmoscope (LSLO) and an AO cSLO (IRIS AO, Berkley, CA) were combined to simultaneously collect complementary* in vivo* images of the primate retina in high-resolution (Figure 2). The AO SD-OCT and cSLO do not have common optics other than the combining beam splitters; therefore, each image plane is collected independently of the other but is recorded simultaneously. The AO SD-OCT [20] and LSLO [21] have been previously described; therefore we describe only the AO cSLO and the integrated setup.

Briefly, the AO SD-OCT portion of the integrated setup provides high-speed (15 frames/sec) cross-sectional scans of the retina with a theoretical axial and transverse resolution of 4 *μ*m and ~10 *μ*m, respectively. The LSLO provides a 30° field of view (FOV) of the retina, which was used to facilitate the alignment of the imager beams on the retina. The AO cSLO system consists of a series of focal telescopes that relay the 7 mm pupil to the optical conjugate planes at the galvanometric scanner, the resonant scanner, the DM (42 actuators MEMS; IRIS AO, Berkley, CA), and the WFS (Adaptive Optics Associates Inc., Cambridge, MA). The pupil is demagnified to 3.3 mm at the plane of the DM and WFS. The resonant scanner frequency is 16 kHz while the acquisition rate of 30 Hz is set by the galvanometric scanner, enabling 512 × 512 images of the retina set to either a 1° or 2° FOV. The AO cSLO beam (632 nm) is input into the combined system through a beam splitter and is reflected off the DM, scanners, and spherical mirrors into the eye. The reflected light follows the same reverse path through the system and onto the WFS and the photomultiplier tube (Hamamatsu H7422-40, Hamamatsu Corp., Japan).

### 2.5. Retinal Imaging

The subject's pupil was aligned to the instrument using the goniometric stage and pupil camera. The retinal features and location of the AO SD-OCT and cSLO probe beam were identified from the LSLO image. Trial lenses were placed at a pupil conjugate in front of the DM of both AO systems to remove lower-order defocus and astigmatism. OCT defocus was removed with the focus adjustment (±5 Diopters) by observing OCT signal intensity and WS spots. Both AO systems were used to simultaneously capture image sequences of the retinal area during laser exposure. Experiments that did not image laser irradiations are denoted by an asterisk in Table 2.

OCT B-scans and cSLO images were collected with and without adaptive optics correction. A line and/or raster scan configuration was used to record OCT B-scans prior to and following laser exposure. Line scans were collected up to a 5.5° scan length on the retina and consisted of 1024 A-scans. Up to 400 B-scan frames were recorded to ensure capture of the longer 413 nm 20-second exposures. Raster scans are multiple line scans swept through the *x* and *y* direction to create volumetric retinal maps and were sized as either 2° × 5.5° or 2° × 2° of retinal area with a B-scan density of 256 or 512. The cSLO frames were recorded in 2° or 1° FOV and collected for duration of 10 or 20 seconds. Prior to laser exposure, images of the macular region were collected using a 2° FOV for the cSLO and a raster scan configuration for OCT. In addition, color fundus photography was performed to provide direct comparison with AO images at each experimental time point.

### 2.6. Lesion Identification and Measurement

Three examiners evaluated all eyes using color fundus photos for each time point following laser exposure. Visible lesions at a given exposure site were reported to be present only if two or more examiners identified a lesion in color fundus photos. Lesion identification in OCT C-scans and cSLO retinal montages of the lesion field was performed by a single person in images with a signal-to-noise ratio greater than 10 dB.

Volumetric OCT images of the macular region were imported into Avizo 6.3 (Visualization Sciences Group, Burlington, MA) for measurement of lesion width and area. Lesions were characterized as regions of hyper- or hyporeflectivity with or without changes in retinal structure in comparison to undisturbed regions. Lesion widths were manually measured as the furthest extent of damage in either the RPE or photoreceptor layer in B-scan images. Lesion areas were selected using a “seed” grayscale pixel value within the hyper- or hyporeflective areas associated with injury. A manually adjusted threshold range based on the grayscale “seed” value was used to select hyporeflective area.

### 2.7. Statistics

The number of visible lesions was determined after 1 hour and 24 hours after exposure, and a probit analysis was performed to determine the dose which corresponds to a 50% probability for damage (ED50). Retinal sites within a single subject receiving multiple exposures of the same laser irradiance were grouped and averaged. Paired *t*-tests were used to compare damage size in the RPE and photoreceptor layers. Lesion widths measured from AO cSLO scans of picosecond exposures were collected from multiple scans of the retinal area.

### 2.8. Pulsed Photothermal Damage: Experiment 1

No lesions were detected below the ANSI MPE limit of 500 *μ*J/cm^2^ for the microsecond laser exposures. Lesions detected in available OCT and AO cSLO images were always observed in color fundus photos. All subjects were reevaluated at the 24-hour observation point with the exception of subject S6. Lesions visible in color fundus photos persisted for all follow-on imaging sessions. Most lesions appeared pale gray to white and transitioned to the former with increasing energy. Exposures which did not result in visible lesions in color fundus photos within the first hour did not develop into lesions at any later time point.

The energy required to create damage for the larger of the two retinal spot sizes (120 *μ*m) required four to seven times greater energy than smaller spot size exposures (40 *μ*m). The range of retinal irradiances producing immediate reflectance changes or visible disruption in either AO SD-OCT or cSLO images was greater for the 40 *μ*m retinal spot size (123–4,563 mJ/cm^2^) than for the 120 *μ*m retinal spot size (72–1,362.4 mJ/cm^2^). The combined ED50 all of microsecond exposures was 495 mJ/cm^2^ and 386 mJ/cm^2^ for the 1-hour and 24-hour observation points (*n* = 80), respectively. Pooling only the larger retinal spot size exposures (*n* = 60), the ED50 value remained unchanged between the 1- and 24-hour observation points (369 mJ/cm^2^).

Real-time* in vivo* imaging of higher energy microsecond exposures revealed disruptive events possibly associated with microcavitation. The AO cSLO acquisition rate of 33 ms/frame is too long to record microbubble formation but can monitor the spatial rearrangement of the retina following exposure. The measured ED50 for immediate retinal rearrangement and/or disruption observed using the AO cSLO in one subject was 826 mJ/cm^2^ (slope of 8.2). This subject received 50 exposures (120 *μ*m spot size) in both eyes ranging from 72 to 1,318 mJ/cm^2^. Visible retinal rearrangement observed in AO cSLO images did not coincide with the appearance of a minimal visible lesion (MVL) in color fundus photos for 10 instances (false negatives). There were no instances in which a disruptive event was observed in an AO cSLO image and did not result in the formation of a MVL in fundus photos (false positive).

**Figure 3:**
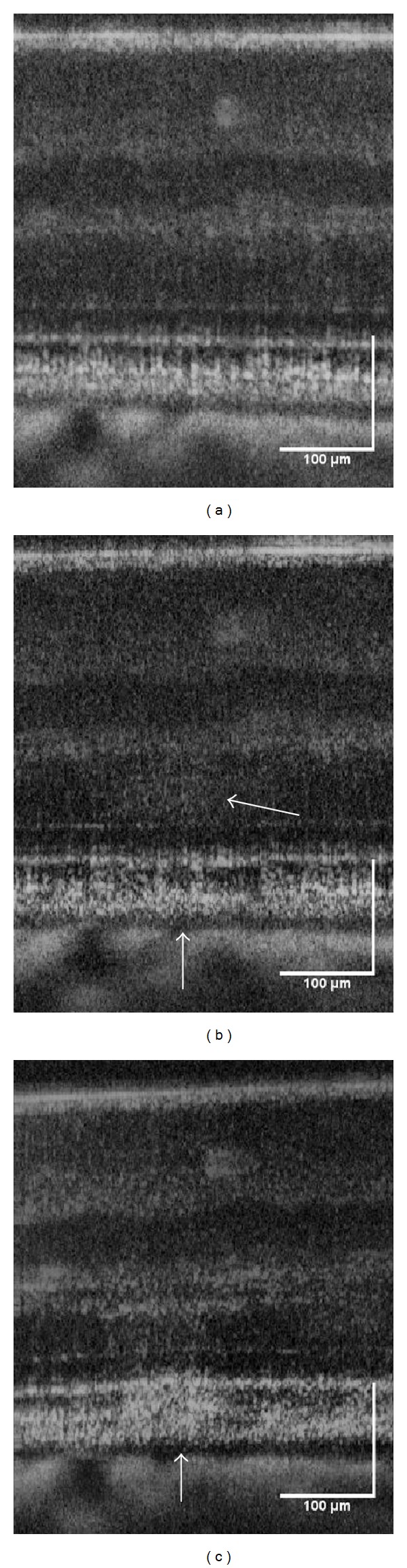
Formation of a lesion in the retina of subject S3 immediately after a high energy retinal radiant exposure (870 mJ/cm^2^). The lesion (white arrows) became more reflective in the minutes following exposure and is likely associated with edema. OCT B-scans of photothermal exposure moments before (a), approximately 10 seconds after (b), and minutes later (c). Scale bar: 100 *μ*m.

Immediately following the higher energy 120 *μ*m spot size exposures, there are slight disturbances to the RPE with increased reflectivity of the outer nuclear layer (ONL) directly above the exposure site.  Figure 3 shows cross-sectional images of the formation of a lesion (870 mJ/cm^2^) in the retina directly before, immediately after, and minutes following laser exposure. Within approximately 10 minutes (Figure 3(c)), the region surrounding the RPE up to the connecting cilia of the photoreceptors appears to swell and there is an increase in reflectivity extending from the RPE into the outer nuclear layer (ONL).

Lesions induced using lower energy exposures (<619.2 mJ/cm^2^) appeared as a mild increase in reflectance of the RPE and ONL in OCT cross-sections and appeared within the hour. The 40 *μ*m retinal spot size exposures occupy a smaller damage footprint compared to the 120 *μ*m spot size exposures (data not shown). “Disruptive” events were not observed for smaller spot size exposures in OCT B-scans or SLO images.

**Figure 4:**
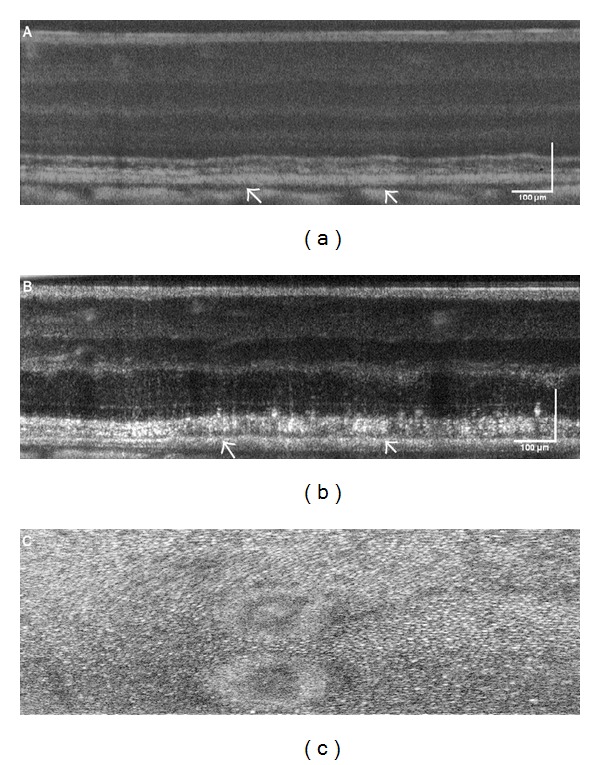
OCT B-scans of a multiple pulse exposure, (1,317 mJ/cm^2^) to the retina of subject S2 OD collected at (a) 1 hour and (b) 24 hours. (c) is an* en face* view of the retina photoreceptor layer of subject S3 OD after receiving a multiple pulse exposure, (874 J/cm^2^). Scale bar: 100 *μ*m.

OCT B-scans of multiple pulse-exposures (1,317 mJ/cm^2^) in subject 2 are shown in Figures 4(a) and 4(b) at the 1- and 72-hour follow-up, respectively.  Figure 4(c) is an* en face* view of the retina photoreceptor layer of subject 3 after receiving a multiple-pulse exposure (874 J/cm^2^).

### 2.9. Photomechanical Damage: Experiment 2

The energy range of 532 nm 40 ps exposures was 6.2–1,843  mJ/cm^2^. Retinal damage immediately visible in AO cSLO images occurred for energies of 139 mJ/cm^2^ and greater. The damage appearance ranged from barely visible retinal disruption sometimes accompanied with reflectance changes to a distinguished “explosive” event. As exposure energy increased, so did the visibility of the disruptive events. Lesion appearance in color fundus photos ranged from pale gray to white increasing in reflectivity and size as energy was increased for each exposure.

None of the 66–221 mJ/cm^2^ exposures in the OS of subject S8 became visible using any imaging modality at any time point. However, exposures ranging from 66–817 mJ/cm^2^ in the OD of S8 observed using AO cSLO produced “disruptive” events or no changes. AO cSLO movies of moderate energy exposures 221–442 mJ/cm^2^ show immediate nonuniform increases in reflectance with or without the appearance of a donut-like “hole” in the lesion center. One hour later, exposures greater than 199 mJ/cm^2^ were visible using both AO imaging modalities. Within a 24-hour period, most exposures became visible in color fundus photos. High energy exposures (>442 mJ/cm^2^) produced few inner retinal hemorrhages and were more readily identifiable at the 1-hour observation points in OCT and AO cSLO images. OCT B-scans collected within an hour of higher energy exposures reveal that the most damage was confined to the outer retina and extended further into the ONL compared to moderate energy exposures. “Explosive” photomechanical disruptions were restricted to exposures of 813 mJ/cm^2^ or greater.

**Figure 5:**
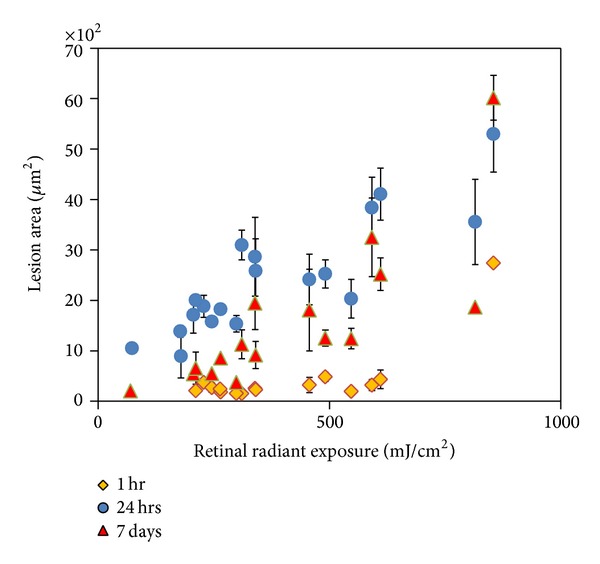
Picosecond lesions area grew (measured using AO SD-OCT) in size proportional to increasing energy measured (data from one subject).

**Figure 6:**
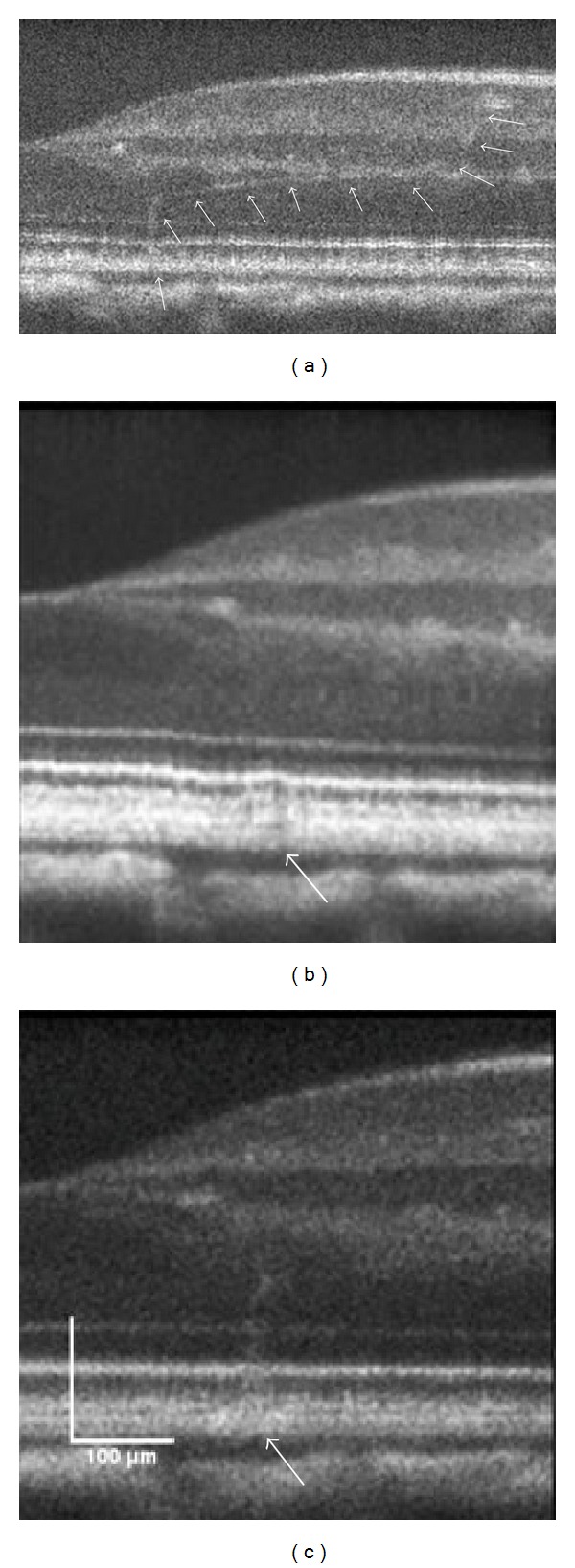
OCT B-scans of the retina of subject S8 OD (a) 1 hour, (b) 24 hours, and (c) 7 days following moderate (325 mJ/cm^2^) 532 nm 40 ps exposure. White arrows in (a)–(c) denote lesion base. Within minutes following exposure, a hyperreflective thread-like structure appears and extends from the lesion site up to a blood vessel (white arrows). Damage to the RPE layer becomes more hyperreflective and distinguishable over a 24-hour period. One week later, the photoreceptor layer protrudes slightly at the exposure site. Scale bar: 100 *μ*m.

**Figure 7:**
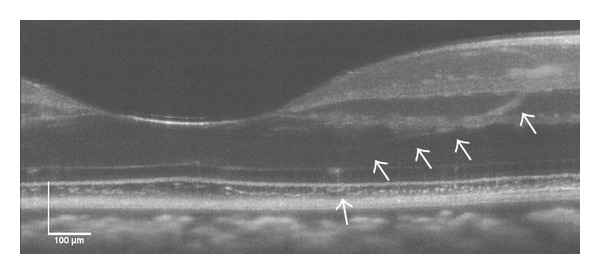
Damage response to picosecond exposure (331 mJ/cm^2^) 1 hour following exposure. Scale bar: 100 *μ*m.

Lesion area increased in proportion to power, as observed from OCT measurements (Figure 5). The entire damage zone surrounding the lesion center is more distinguishable in cSLO images at the 1-hour observation time point in comparison to OCT* en face* and cross-sectional images. Observations of the lesion area using cSLO allowed viewing of a definite hyperreflective region sized approximately 60–120 *μ*m in width with a hyporeflective center. OCT cross-sections reveal a punctate increase in hyperreflectance extending from the RPE to the ONL (Figures 6 and 7). Lesion widths measured from OCT* en face *images of the photoreceptor inner segments ranged from 20 to 40 *μ*m. The lesion area measured from OCT* en face* images at the 24-hour follow-up suggests that the lesions increase in size from 29 to 50%. Lesion area decreased by 2–41% in the week following exposure.

### 2.10. Photothermal/Photochemical Damage: Experiment 3

**Figure 8:**
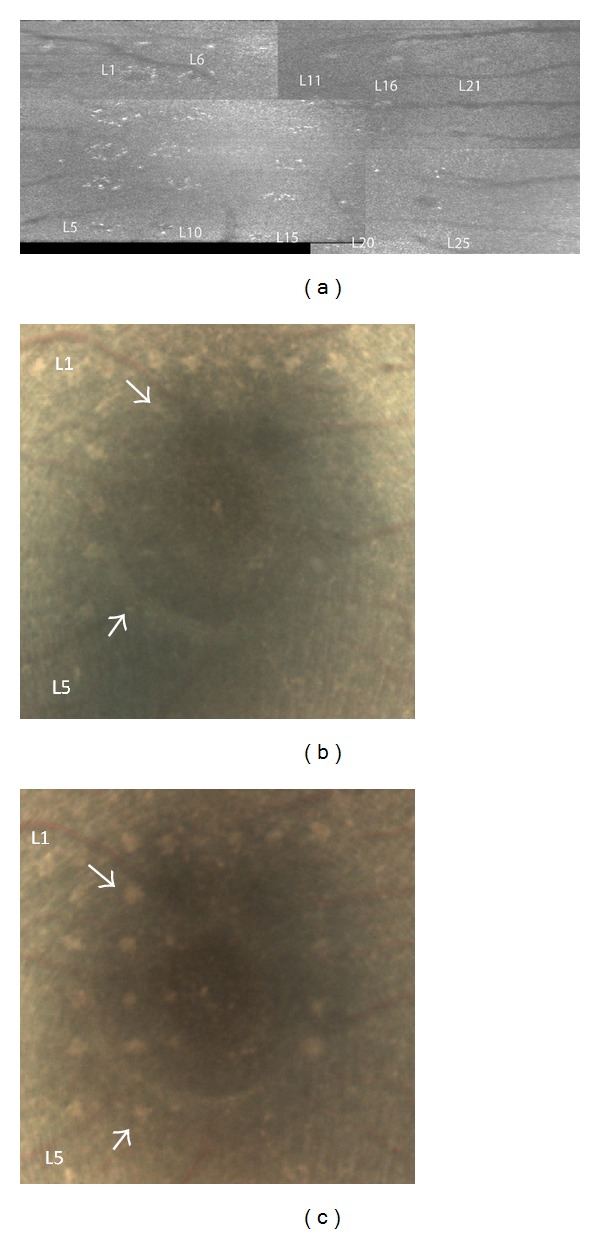
(a) OCT* en face* images collected 1 hour after exposure of retinal area receiving 413 nm light insult (41–1,089 J/cm^2^). (b) Color fundus image of same retinal area shown in (a) 1 hour after exposure. (c) Color fundus image of same subject 10 days after exposure.

The retinal sites exposed to 413 nm light had a pale yellow appearance in color fundus photos, which was maintained through all observation time points. For all subjects, retinal damage observed using SD-OCT associated with laser exposure was not always positively identified as MVLs in color fundus photos. Of the total 173 lesions, there was a 46% improvement in lesion detection using OCT* en face* images (*n* = 93) versus color fundus photos (*n* = 43) at the 1-hour time point. In Figure 8, of the 25 retinal sites receiving 413 nm exposures ranging from 165 to 1,183 J/cm^2^, 24% (7 out of 25) qualified as MVLs in color fundus photo taken 1 hour later even though hyperreflective spots coincident with exposure locations can be seen in the OCT* en face* view (Figure 8(a)). OCT C-scans of the photoreceptor inner segments reveal that 88% (22 out of 25) of the exposed regions are inhomogeneously hyperreflective. Ten days later, 56% of the lesions were identified as MVL in color fundus photos (Figure 8(c)) compared to 80% identified using OCT raster scans.

**Figure 9:**
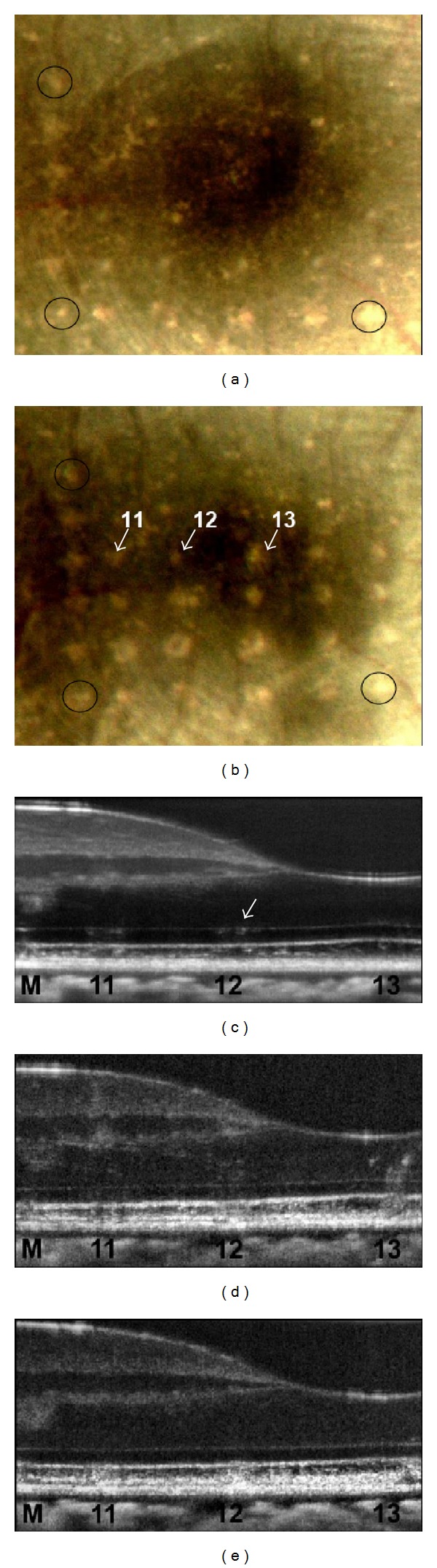
Fundus and OCT B-scans of 413 nm exposures in the retina of one subject. (a) Lesions 11–13 (474 J/cm^2^) were not positively identified as MVLs in color fundus photos at the 1-hour observation point. (b) At the 24-hour observation point, all three exposure sites were positively identified as a MVL. OCT B-scans collected within minutes of the initial exposure (c) reveal a hyperreflective “clumping” in the photoreceptor inner segment layer. The RPE layer shows a modest increase in reflectance with a “granular” appearance. (d) The damage surrounding the RPE and photoreceptors becomes more distinguishable 24 hours later. A wispy “threading” at the boundary of the inner nuclear layer (INL) and outer plexiform layer (OPL) for lesions 11 and 12 becomes observable. An increased reflectance of the inner retinal layers directly above the lesion site suggests an edematous reaction. Nine days later, (e) the damage appears more homogenous and extends from its base at the RPE into the connecting cilia of the photoreceptors.

OCT B-scans and cSLO images collected during 413 nm, 20-second exposures did not reveal any immediate changes to the retina during laser irradiation. Exposures greater than 162 J/cm^2^ produced hyperreflective changes in retinal OCT B-scans collected within 1 hour following exposure. Retinal layers affected were the photoreceptor inner segments, RPE, and inner retina.  Figure 9 shows the retinal damage response of three retinal locations receiving a retinal exposure of 474 J/cm^2^.

**Figure 10:**
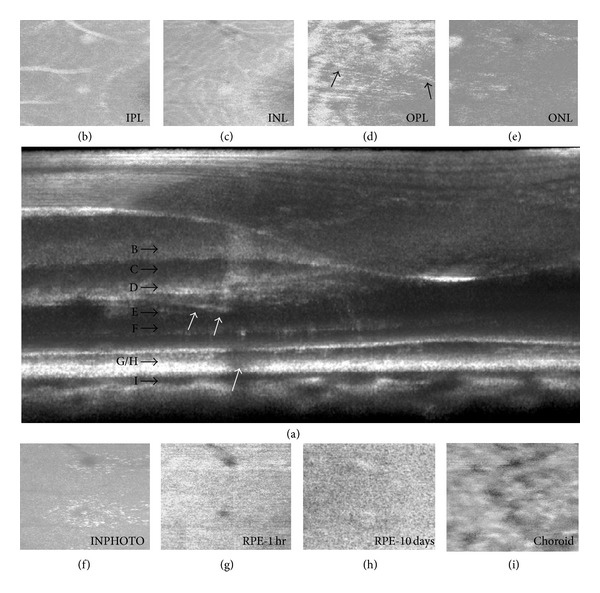
OCT B-scan and en face fly-through of subject S6 retina after receiving 413 nm (240–1,183 J/cm^2^) exposures 1 hour (a)–(g), (i) and 10 days later (h).

**Figure 11:**
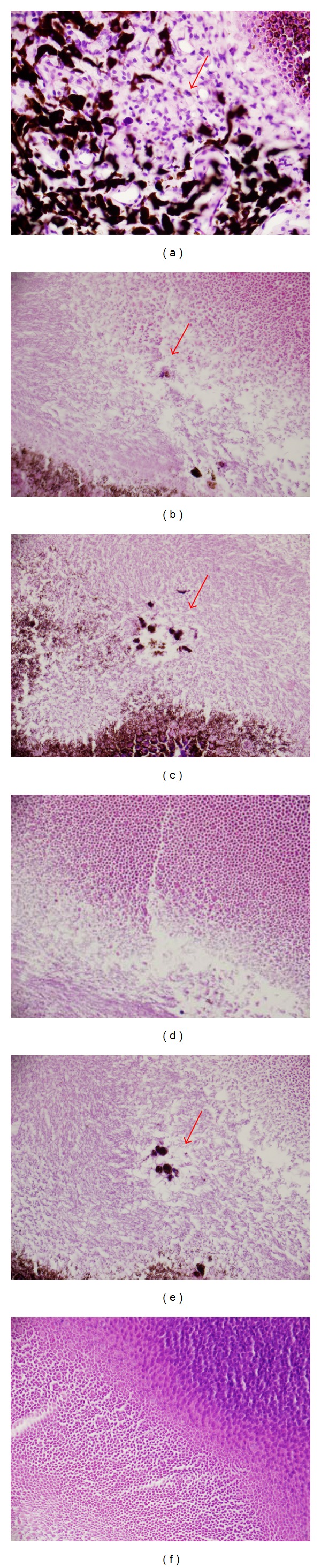
Subject S6 OD histological sections of a flat mount retina nine days after exposure (1,696 J/cm^2^). (a) Choroid, (b) outer segment of photoreceptors, (c)-(d) junction of inner and outer segment of photoreceptors, (e) inner segment of photoreceptors, and (f) junction of inner segment and outer nuclear layer. The choroid exhibits loss of pigment as well as low numbers of macrophages, lymphocytes, and plasma cells. There is disruption of the outer segments of the photoreceptors and pigment laden macrophages (b). (c) and (d) both show the junction of inner and outer segment of photoreceptors with minimal disruption of photoreceptors and two small pigment laden macrophages. The inner segment of photoreceptors with minimal disruption of photoreceptors (linear defect is an artifact) is shown in (e). The final frame (f) is the junction of inner segment and outer nuclear layer with no detectable defect.

Figure 10 is a 1-hour OCT retinal cross-section of a 1,696 J/cm^2^ exposure with complementary* en face *views. The damage extends full thickness but does not have a uniform appearance as it extends from the retinal ganglion cell (RGC) layer into the RPE.  Figure 11 shows histological sections from a retinal flat mount of the same lesion nine days following exposure.

**Figure 12:**
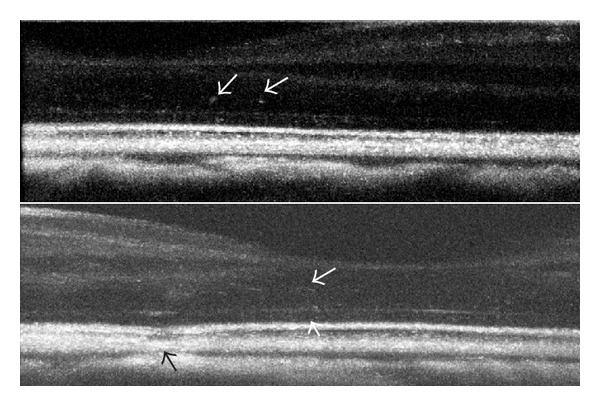
Projection view of OCT cross-sections of 413 nm exposures (24 hours) not visible in color fundus photos at the 1-hour or 24-hour time point. Retinal exposures ranged from 225 to 1,089 J/cm^2^. Lesions eventually became visible in color fundus photos 3 days later. Note the hyperreflective regions and threading present (white arrows). Black arrow denotes thermal marker lesion.

Lesions which did not become visible in color fundus photos until the 72-hour observation point had retinal irradiance values ranging from 225 to 1,089 J/cm^2^. OCT and cSLO scans captured during exposure and 1 hour after exposure did not present any changes in retinal structure or reflectance. Raster OCT scans collected 24 hours later revealed 10–15 *μ*m round hyperreflective structures within the ONL closest to the fovea with appearance of “threading” extending from “round body” like structures (Figure 12). No changes were seen in cSLO images.

**Figure 13:**
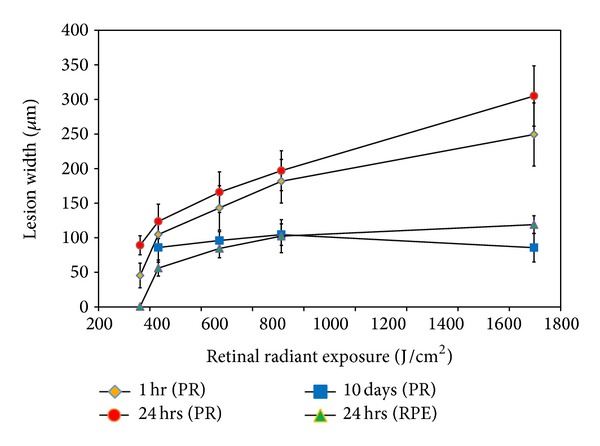
Plot of lesion width versus retinal radiant exposure to 413 nm light for one subject. Measurements were collected from AO SD-OCT* en face *images at the RPE and photoreceptor layer at 1 hour, 24 hours, and 10 days after exposure. PR: photoreceptor layer; RPE: retinal pigment epithelial layer.

Minimal or no damage was visible to the RPE for exposure energies less than 1,696 J/cm^2^ in OCT C-scans for the first hour after exposure; therefore, lesion width measurements were collected only at the photoreceptor layer for the first hour where retinal changes were visible. Over the course of 24 hours, the damage zone of the photoreceptor layer significantly increased in size (*P* = 0.02) for one subject (Figure 13). Damage at the 1-hour time point for the remaining subjects was not clearly demarcated or subjects did not have a 24-hour follow-on imaging session. The differences in lesion size between the photoreceptor layer and RPE at 24 hours for a single subject were significantly different (*P* = 0.02) (Figure 13). Overall, the lesions decreased in size beyond the 48-hour observation period.

The ANSI MPE calculations based on the experimental parameters provide a limit for both thermal and photochemical exposures. For this set of experiments, the limits for thermal and photochemical damage are 57.2 and 2.44 J/cm^2^, respectively. Exposures at the 1-hour time point which are greater than the lower limiting photochemical MPE were identified as inducing retinal reflectance changes in OCT and fundus images for 62% and 42% of the exposures, respectively. There was an improvement in lesion identification using fundus photos 48 and 72 hours after exposure when compared to the number of lesions identified using OCT. In particular, for subject S7, none of the 15 lesions above the thermal limit was identified using fundus images at the 1- and 24-hour observation time points but was observed 72 hours later (73%). Lesions were identified (27%) at the 1-hour observation time point from OCT* en face* images.

## 3. Discussion

The introduction of high-resolution retinal imaging into light damage studies has enabled greater detection of the effects of laser light exposure compared to traditional methods employed in past studies of laser damage thresholds. Progress in understanding the mechanism for laser interaction with retinal tissue is driven both by advances in ophthalmic laser therapy and by the need to establish mechanistic underpinning for laser eye safety standards. For the first time, to our knowledge, we have been able to image the immediate effects of photomechanical disruption to the retina.

The data presented within this study are a pilot study using high-resolution imaging to study laser-tissue interactions* in vivo*. Short pulse laser exposure can damage the retina via photomechanical or photothermal mechanisms depending on the pulse duration and total energy delivered. Real-time* in vivo* imaging of higher energy microsecond exposures revealed disruptive events likely associated with explosive vaporization of water and subsequent cavitation around melanin particles in the RPE cells. The transition from thermal denaturation into thermomechanical effects as the primary cause of damage to the RPE after laser exposure is approximately 50 *μ*s [22]. Schuele et al. demonstrated in experiments utilizing* ex vivo *porcine RPE samples that microbubble formation is the dominant damage mechanism to the RPE for 5 *μ*s pulse durations. Photomechanical events possibly associated with microcavitation disruption may have been observed at the greater end of exposure energies for Experiments 1 and 2 but in all likelihood were mostly thermal in nature. Previous studies have used reflectometry [22, 23], stroboscopic imaging [24], and acoustic measurements [25, 26] to monitor microbubble formation. The AO SLO acquisition rate of 33 ms/frame does allow recording of actual microbubble formation (~20 *μ*s) on the order of microseconds but can monitor the spatial rearrangement of the retina following exposure. Although our experiments did not use any complementary independent methods, such as reflectometry, other than OCT cross-sections to confirm the presence of microbubbles, the 2 *μ*s pulse duration used in our studies was likely associated with thermal damage. The ED50 threshold for detecting visible disruption from 2 *μ*s in AO cSLO images was 827 mJ/cm^2^. The threshold for microbubble formation in* ex vivo* porcine RPE reported by Schuele et al. [25] was 222 mJ/cm^2^. Beam profile distributions and size (40 *μ*m versus approximated 120 *μ*m retinal spot size) could account for the difference in threshold. The false negatives for damage resulting from the measurement of the spatial disruption of the retinal layers as an indicator of bubble formation indicate that thermal denaturation may still be the critical damage mechanism for the laser parameters tested. However, the presence of retinal layer disruption should be a clear indicator of a mechanical process and thus this compels the belief that 2 *μ*s is close to the exposure time associated with the transition of damage from photothermal to photomechanical mechanisms. This observation is consistent with theoretical model results of laser induced damage processes in the retina [27]. While false negatives of retinal layer disruption as an indicator of bubble formation suggest thermal denaturation as the predominate damage mechanism at threshold, other possibilities for the lack of physical evidence of microbubble formation include obscuration by imaging artifact and low signal-to-noise ratio in SLO imaging. Furthermore, slight misalignment of the insult and OCT beam may have resulted in B-scans recorded to be slightly adjacent to the site of highest irradiance during exposure.

None of the exposure levels was below the MPEs set by ANSI for respective laser parameters. The retinal effects of microsecond and picosecond laser pulses are dependent on the energy per pulse and laser spot size at the retina. Lesion size and area of 3-picosecond exposures were logarithmically related to the amount of energy delivered to the retina. For the microsecond exposures, there was retinal spot size dependence when comparing energies required for creating MVLs as well as the extent of injury. The radiant exposure needed to create damage is greater for the larger spot but results in a more exaggerated disruptive event in comparison to the smaller retinal beam size. One reason may be due to a larger volume of fluid being heated surrounding the increased number of melanosomes within the irradiated area. As a result, the formation of larger bubbles due to coalescence of multiple smaller bubbles could cause an exaggerated “explosive” disruption.

Using a laser with an annulus and top hat beam distribution profile, Kennedy et al. 2004 [28] show that the ED50 threshold for 3 us 590 nm pulses varies with retinal spot size. Our results followed a similar trend in which more energy is required to produce damage with a larger retinal spot size and point to thermal mechanisms at play. Previous picosecond laser damage studies [14, 29] have compared the extent of damage and damage thresholds to rhesus monkey retinas based on spot size differences. The energy range of picosecond exposures at the cornea for this set of experiments 6.2–1,843 mJ/cm^2^ (0.03 to 8.34 *μ*J at the cornea) was less than the exposure energies to rhesus monkeys in the study by Roach et al. [14] (4–20 *μ*J at the cornea). The differences in beam size and two different primate species do not allow direct comparison of damage characteristics; therefore, we will only compare damage trends. In addition, picosecond image data were retrieved in only one subject. Low energy exposures in subject S7 did not produce ophthalmoscopically visible damage. Color fundus photos from subject S8 were out of focus due to corneal aberrations and therefore did not allow inclusion for probit analysis. The energy spread of picosecond exposures of subjects S7 and S8 resulted in all positive MVLs. As a result, the energy distribution of applied exposures and number of eyes available for use in probit analysis (*n* = 3) were not sufficient.

Results of aforementioned picosecond studies are similar in regard to the localization of damage in the outer retina when laser induced breakdown (LIB) does not play a role. The experimental irradiances were not great enough to produce LIB in the primate retina [30]. In addition, as the energy of the exposure increases, the extent of the damage also increases to include the inner retinal layers. The “collapsed” appearance of the photoreceptor layer following moderate to high power exposures suggests explosive mechanisms. Low signal-to-noise ratio, frame rate, poor light transmission, eye movement, and misalignment of the imaging laser with insult laser may account for missed and unobserved photomechanical phenomena* in vivo.*

### 3.1. Photothermal/Photochemical Damage: Experiment 3

The retinal radiant exposures used in this set of experiments were 40.4–7,000 mJ/cm^2^. The dominant light induced damage for this set of experiments is primarily driven by photochemical mechanisms. It is possible that the damage mechanism transitioned from a photochemical to thermal effect or was partly thermal and photochemical in nature. Probit analysis of 413 nm data failed to converge to a solution for either 1- or 24-hour observations and may point to small differences in energy required to transition between damage mechanisms. Not all 413 nm damage observable using high-resolution imaging modalities was noted as an MVL in color fundus photos. For example, in more than one subject and multiple eyes receiving 413 nm 20-second exposures, damage was visualized at the one-hour time point in OCT B-scans as small nonuniform regions of “speckled” hyperreflectivity at the RPE and photoreceptor layers, but damage was not identified in color fundus photos. In addition, lesions from one subject were not visible in color fundus photos until the 72-hour time point despite evidence of light induced changes in OCT scans at earlier time points. The time course of photochemical lesion development to become funduscopically visible is on the order of 2-3 days after exposure [31, 32]. It is possible based on our findings that observable retinal changes associated with photochemical damage could occur on a much shorter time scale (hours) as compared to days but are not observed using traditional fundus photography. In another study investigating possible photochemical/photothermal damage [3], a reduction in RPE fluorescence occurred immediately following 900 s 568 nm laser exposures with an average power greater than 47 *μ*W. It is not known whether the exposures were funduscopically visible 1–3 days following injury as color fundus images were not collected until 6 days following injury. The majority of retinal exposures used to study 413 nm damage in this study are greater than 47 *μ*W and within ANSI standards for the MPE at the lower limit photochemical light exposure. Exposures not within the limit did not present any observable retinal changes.

The 413 nm 20 exposures closest to the fovea with energies greater than 700 J/cm^2^ resulted in full retinal thickness reflectance changes associated with damage and edematous reactions. The 413 nm wavelength falls within the macular pigment absorption band [33]. The macular pigment concentration is greatest at the foveal pit center and decreases exponentially out to approximately seven degrees eccentricity from the pit center. Most of the macular pigment is localized within the Henle Fibers, IPL and OPL [34]. The macular pigment protects the retina from blue light damage [35] as it serves as a blue light filter and antioxidant. Previous studies [36, 37] have demonstrated the difference in lesion localization with respect to the fovea and examining injury from argon and krypton laser wavelengths. Marshall and Bird [37] examined 50–300 mW argon laser exposures from 1° up to 10° eccentricity from the foveal center. Argon lesions delivered outside of a 2° circle of eccentricity from the foveal pit were localized to the RPE while lesions within the 2° circle extended from the RPE into the inner retina and appeared “full-thickness.” The localization of damage described here closely resembles the histological analysis of argon laser damage to the fovea and its periphery. The ability to follow lesion progression over several time points after exposure (up to 9 days) revealed the “full-thickness” damage we observed at 1 hour and 24 hours after exposure is no longer visible in OCT at a 9-day follow-up point. Histology of those same lesions also did not show any observable damage within the inner retinal layers.

The damage response following exposure for the 413 nm 20-second and 532 nm 40-picosecond exposures was observed using AO SD-OCT. The “thread-like” structures develop within the hour following exposure and are localized to the lesion site at the external limiting membrane (ELM) and reach into the OPL and are generally seen beyond the foveal region but is limited to lesions closest to the fovea. Lesions closer to the fovea display higher density “threading” than those further away. The filamentous structures were not observed at the 1- or 24-hour observation point for the 570 nm 2 *μ*s exposures but did appear following picosecond exposures but not as densely. At the 24-hour observation time point, the “threading” increases in reflectivity and size. A possible explanation includes damage to the Henle Fiber layer due to transsynaptic degeneration and direct damage from laser exposure [36].

Müller cells play a key role in the retinal response to injury. In particular, light (488 nm) injury is associated with enlargement of Müller cell processes, gliosis [38], and upregulation of GFAP [39]. GFAP intermediate filaments are upregulated and endoplasmic reticulum is increased. GFAP upregulation in Müller glial cells following retinal injury and disease is the most “sensitive nonspecific” response to cellular dysfunction and can be used as a “retinal stress indicator” [40]. Such changes could cause an increase in the refractive index of the Müller cell cytoplasm, with increased visibility of the structure by high-definition OCT. The “threading” spans from the external limiting membrane (ELM) and to the OPL. The Müller cells extend from the ELM inward to the internal limiting membrane (ILM) and can extend their foot processes instead to rest upon intraretinal blood vessels where they form part of the blood-retinal barrier [40, 41]. The cone axons of the Henle fiber layer are “bound” and encased by the outer processes of Müller cells and both run in parallel as they leave the central foveal area [42]. The number of Müller cells to cone terminals in the perifoveal OPL is equal, and the Müller cells per/mm^2^ decrease with increasing distance from the fovea [43]. This would partially help to explain the reduced appearance of the threaded structures away from the fovea.

Another possible explanation for the threading process is the accumulation of serum or blood leakage from laser-damaged intraretinal vessels; such vascular damage with a 488 nm laser has been illustrated [36, 37]. The serum or blood would tend to infiltrate the retina along the path of least resistance, in spaces formerly occupied by intact Müller cells. Although this idea could explain the time frame of our observations, the very sharply defined quality of these reflective thread-like structures does not appear similar to blood vessel hemorrhage observed in OCT B-scans after pulsed laser exposure [14].

## 4. Conclusion

This is a pilot study to examine the feasibility of using AO SD-OCT and AO cSLO to examine regimes of laser damage in real-time in addition to the damage response. There is an increased detection of blue light damage at the 1-hour time mark using high-resolution imaging prior to lesion appearance in fundus photos at 24 hours and later. The results highlight the need for continued use of high-resolution imaging to resolve retinal light damage in support of laser safety guidelines. With regard to pulsed thermal laser exposure, high-resolution imaging systems with high frame rate may produce more accurate representation of the pulsed laser tissue phenomena. Future studies should include histology to explain and interpret the filamentous threaded structures emanating from the damaged region.

The AO SD-OCT and cSLO provide researchers with high-resolution images of retinal layers to resolve laser damage* in vivo*. This approach also provides an opportunity to observe laser damage over multiple time points reducing the need for histopathology as a single end point for observation. Future studies should further examine regimes of light damage exposures below the MPE. AO systems are developed and refined for clinical operation; they will continue to improve the ability of the ophthalmologist to visualize diseased or damaged tissue and manage therapies.

## Figures and Tables

**Table 1:** Summary of exposure parameters.

Experiment	*λ* (nm)	*τ*	Retinal spot (*μ*m)	Energy at cornea	Retinal radiant exposure	Trials
1	555	2 µs	120 and 40	(8.1–155 µJ) (1.6–57 µJ)	72–4,536 mJ/cm^2^	*n* = 8
2	532	40 ps	24	0.03–8.34 µJ	6.2–1,843 mJ/cm^2^	*n* = 4
3	413	20 s	105	3.5–606 mJ	40.4–7,000 J/cm^2^	*n* = 4

**Table 2:** Summary of imaging schedule for experiments 1–3.

Subject	Age	Experiment	Eye	Imaging schedule
S1	4	3	OS	1 HR 6 D
S1	4	3	OD	1 HR 7 D
S2	4	3	OS	1 HR 2 D
S2	4	3	OD	1 HR 24 HR 3 D
S3	5	1	OD	1 HR 24 HR
S3	5	1*	OS	1 HR 24 HR 10 D
S4	4	1*	OS	1 HR 6 D
S4	4	1	OD	1 HR 24 HR 7 D
S5	5	1*	OS	1 HR 48 HR
S5	5	1	OD	1 HR 24 HR 3 D
S6	4	1	OS	1 HR 9 D
S6	4	1*	OD	1 HR 24 HR 10 D
S7	11	2	OS	1 HR 48 HR
S7	11	2	OD	1 HR 24 HR 3 D
S8	5	2	OS	1 HR 6 D
S8	5	2	OD	1 HR 24 HR 7 D
